# Virtual reality exergame in older patients with hypertension: a preliminary study to determine load intensity and blood pressure

**DOI:** 10.1186/s12877-023-04245-x

**Published:** 2023-08-30

**Authors:** Susan Vorwerg-Gall, Oskar Stamm, Michele Haink

**Affiliations:** Department of Geriatrics and Medical Gerontology, Charité – Universitätsmedizin Berlin, corporate member of Freie Universität Berlin and Humboldt-Universität zu Berlin, Reinickendorfer Straße 61, 13347 Berlin, Germany

**Keywords:** VR training, Exergame, Head mounted display, Physical activity, Postexercise hypotension, Heart rate, hypertension, High blood pressure, Older adults

## Abstract

**Background:**

Lifestyle changes and physical activity can make an important contribution to reducing the risk factor for high blood pressure (BP). Whether virtual reality (VR) exergames are also appropriate and make a positive contribution to the reduction of BP has not yet been sufficiently investigated. Therefore, the aim of the study was to gain knowledge of the load intensities to be achieved during a VR exergame and to examine the short-term effects on BP.

**Methods:**

For the preliminary study, 22 participants with hypertension over the age of 65 years were analyzed. The study took place in a mobile laboratory truck. All participants visited on two occasions. During visit 1, VR strength endurance training (VR-SET) and during visit 2, VR endurance training (VR-ET) was performed. Each VR session lasted approximately 25 min and was of a moderate intensity. Heart rate (HR) was measured across the entire session, as well as BP before and after the VR exergame. The Rating of Perceived Exertion (RPE) and task load using NASA Task Load Index were determined after each VR session. Included in the statistical analysis were the Shapiro–Wilk test, the paired t-test, the Wilcoxon test and ANOVA for repeated measures.

**Results:**

During the “main part” (*p* < .001), at the “end” (*p* = .002) and for the “maximum HR” (*p* < .001), significant load differences between both VR sessions could be determined. In addition, significantly more participants in the VR-SET group achieved a moderate load intensity of at least 40% of heart rate reserve (*p* = .014). Regarding RPE, participants rated their subjectively perceived exertion significantly higher in the VR-SET than in the VR-ET (*p* = .028). Systolic BP decreased significantly in both VR sessions when compared before VR session and 5 min after VR session (*p* = .015; *p* = .003), as well as before VR session and 10 min after VR session (*p* = .018; *p* < .001).

**Conclusions:**

An individual moderate load intensity of 40% can be reached during VR-SET. In addition, a positive short-term effect of the VR exergame on BP behavior (postexercise hypotension) was observed after both VR sessions. The preliminary results indicate that a VR exergaming could lead to blood pressure lowering effects for older people with hypertension.

**Trial registration:**

The study was registered in the German Clinical Trials Register (DRKS-ID: DRKS00022881, 07/09/2020, https://drks.de/search/de/trial/DRKS00022881).

## Introduction

With more than 10 million deaths per year, hypertension is one of the most common preventable causes of death worldwide and is one of the most important risk factors for cardiovascular diseases (CVDs) [1, 2]. Especially in the over 60 age-group, approximately two thirds of these individuals suffer from hypertension [2, 3].

However, hypertension can be influenced by individual health behavior. Lifestyle changes, such as dietary changes, restrictions on alcohol and tobacco consumption and above all physical activity, can lead to antihypertensive effects and minimize cardiovascular risk factors [4]. In particular, the positive influence of physical activity on blood pressure (BP) has been demonstrated by various epidemiological studies [5–7]. A meta-analysis of RCTs has shown that aerobic endurance training, dynamic resistance training and isometric training reduce resting systolic BP/diastolic BP by 3.5/2.5, 1.8/3.2 and 10.9/6.2 mmHg, respectively [6]. According to these studies, a combination of aerobic exercise and resistance training in older individuals provides the best effect in lowering BP, increasing arterial elasticity and thus reducing the risk of CVD [5]. In this regard, guidelines from the American College of Cardiology (ACC)/American Heart Association (AHA), International Society of Hypertension (ISH) or the European Society of Cardiology (ESC)/European Society of Hypertension (ESH) recommend that individuals with hypertension should perform at least 30 min of dynamic aerobic exercise (walking, jogging, cycling or swimming) at a moderate intensity five to seven days per week, as well as resistance exercise two to three days per week [8–10]. In Germany, the recommendations are not being regularly achieved [11]. Approximately 70% of people over the age of 65 are physically active for less than 2.5 h per week [11]. In this context, exergames could make an important contribution to motivating older adults to become more physically active. The increased motivation aspect has been confirmed in studies and has also resulted in an increase in well-being and enjoyment [12–14]. Exergames has also been used successfully as a tool to facilitate physical activity in older people [15–17]. However, there is limited research on exergaming and hypertension in older adults. Studies involving young adults/students who examined BP during exergaming, identified positive effects on BP [18–20]. Hypertension was not previously considered a defined inclusion criterion and could not be found in any previous studies with exergames [21]. Furthermore, there is insufficient evidence that whether a VR exergame is capable of achieving the required moderate load intensity in older adults with hypertension. In this context, a study by Silva et al., involving adult students, indicated that on average, lower HR values of below 90 bpm were achieved during VR exergaming [20]. In the context of older participants, very different results from 80 to 125 bpm could be determined [22–24]. However, maintaining the intensity for optimal training and thus avoiding over- and underload was crucial to achieving positive health effects among older patients with hypertension [25].

Consequently, the aim of the study was to investigate the load intensity and BP of older patients with hypertension during a VR exergame. To achieve this goal, the following primary research question was to be answered: Is the load intensity of the VR exergame appropriate, so that older patients with hypertension can exercise within their individually calculated moderate load intensity of 40% to 60%and could changes in BP values (systolic, diastolic, pulse) be detected after the VR exergame?

## Methods

### General

The registered study was a preliminary study evaluating a VR exergame (German Clinical Trials Register 07/09/2020, DRKS-ID: DRKS00022881). Data were collected prospectively during two visits. All participants were recruited through the volunteer database of the Geriatrics Research Group of the Charité Berlin, certain sports centers and geriatric organizations. There were no personal relationships between the study team and the participants. To recruit potential participants, telephone calls were made, and e-mails with study information were sent (*n* = 174). For the study, 32 potential participants over the age of 65 years who had been diagnosed with essential hypertension were recruited and checked for inclusion and exclusion criteria (except the test for risk of falls) by telephone (Table 1). To check the cognitive status the TICS-Telephone Interview for Cognitive Status [26] was used.

**Table 1:** Inclusion and exclusion criteria of the study

Inclusion criteria	Exclusion criteria
**•** ≥ 65 years **•** Diagnosed essential hypertension (stage I), self-report of the medical diagnosis **•** Independent mobility **•** Presence of a signed informed consent form	**•** Presence of legal guardianship **•** MCI and severe cognitive impairment (TICS < 33 points) **•** Dizziness **•** Severe visual impairment **•** Motion sickness **•** Medical conditions associated with high risk of falls (stroke, Parkinson's disease) **•** Increased risk of falling tested on site before visit 1 (Tinetti test < 24 points)

*TICS* Telephone Interview for Cognitive Status

**Fig. 1:**
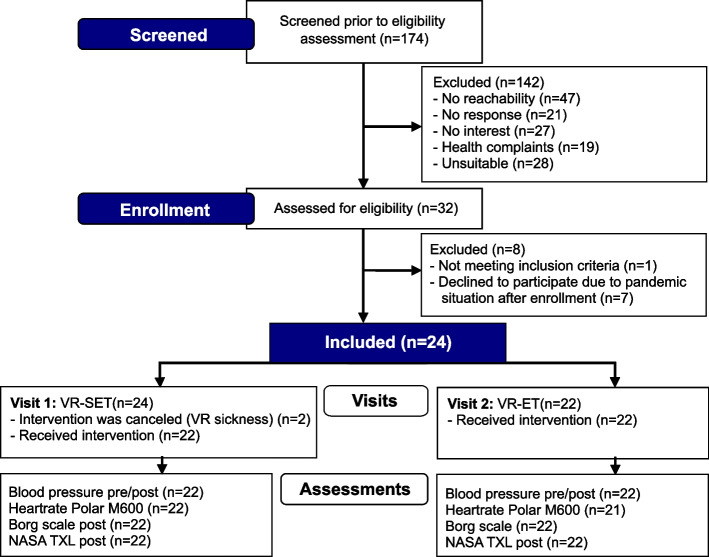
Flow diagram of the recruitment process, visits and assessments

However, due to the worsening pandemic situation, seven of the participants recruited withdrew from the study. A total of 24 participants took part. Two participants dropped out of the study due to VR sickness symptoms at the beginning of the first VR session (Fig. 1).

**Fig. 2:**
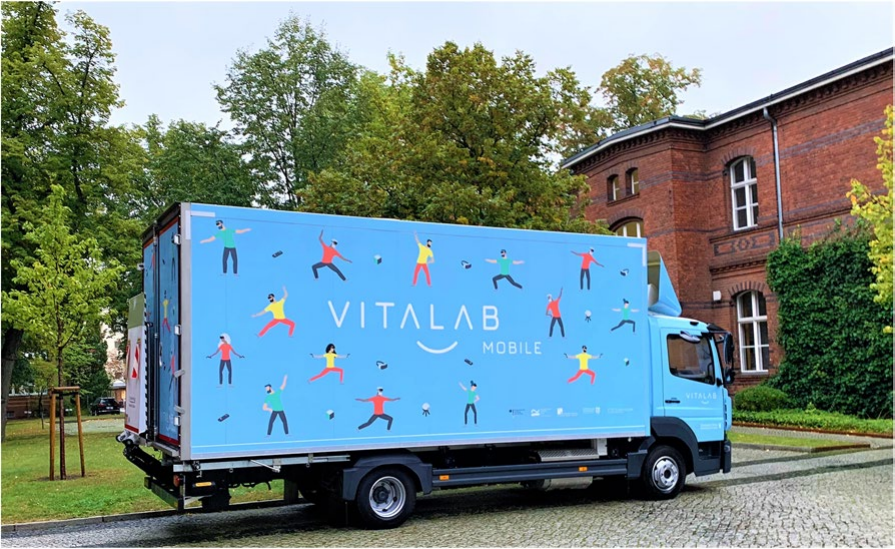
Mobile research laboratory VITALab.mobile

The study took place in the mobile research laboratory VITALab.Mobile [27] in the form of a truck on the campus of the Evangelisches Geriatriezentrum in Berlin (Fig. 2). During the study period from 10–05-20 to 11–06-20, the participants made two individual visits within two weeks. Each participant tested the VR-SET on week one (visit 1) and the VR-ET on week two (visit 2). The study was organized, supervised and conducted by two research assistants with a background in physical therapy and one trained public health master's student. The study design did not include randomization and blinding of participants and study personnel. After all included participants completed visit 2, the study was terminated.

### Intervention design

On visit 1 the Tinetti test [28] was performed to eliminate the exclusion criteria “risk of falling”. Also framework data were collected, and a BP measurement was taken three times before the VR session. Therefore, the BM28 HSD upper arm BP monitor from Beurer GmbH was used. During both VR sessions the heart rate was measured with a smartwatch.

**Fig. 3:**
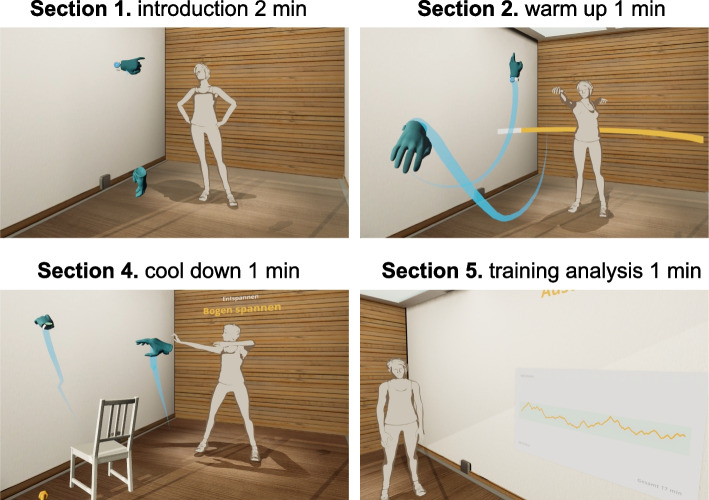
Sections 1, 2, 4 and 5 of the VR Exergame

The VR session, lasted between 20 and 25 min and was divided into five sections: 1. introduction by the virtual trainer agent named “Anna”; 2. warm up; 3. main part; 4. cool down; and 5. training analysis. Only Sect. 3, “main part”, differed between the two VR sessions. The “main part” focused on moderate intensity (40–60% of heart rate reserve (HRR)) according to the recommendations of the American College of Sport Medicine (ACSM) for patients with hypertension [29]. The other four sections remained identical across both visits (Fig. 3).

**Fig. 4:**
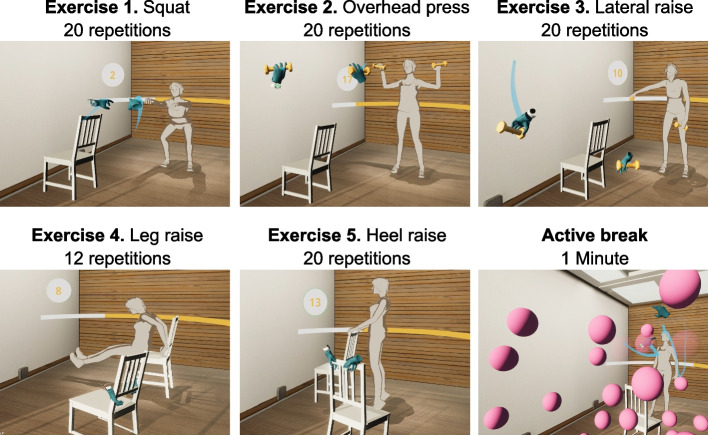
Section 3 -”main part” of the VR-SET (visit 1)

The “main part” of the VR-SET was divided into a learning phase and an exercise phase and lasted approximately 15 to 20 min. For the VR-SET, a chair and dumbbells were used as training objects. In the learning phase, the participants performed the five exercises three times (Fig. 4). During the exercise phase, these five exercises were executed in two sets. Each exercise was repeated 12 to 20 times. Between the exercises was a short break of 20 s. An active break of 1 min took place after the first set. Within this time, the participants had to make as many colored balls as possible vanish by touching the balls with their hands.

**Fig. 5:**
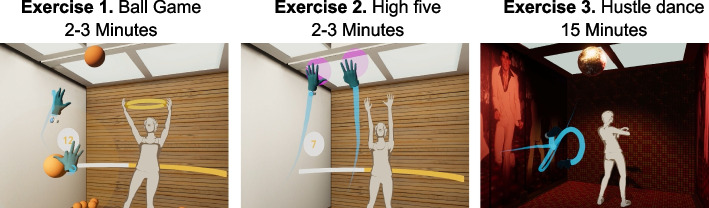
Section 3 -”main part” of the VR-ET (visit 2)

During the “main part” of the VR-ET, three exercises were completed (Fig. 5). In the “ball game”, a ball was grasped with either the left or the right hand and thrown into a ring. After three hits, Anna changed the position of the ring. During the “high-five” exercise, Anna's movements had to be mirrored, which involved touching two pink balls with both hands at the same time. When this was mastered successfully, Anna changed the position. During the “dancing” session, the VR environment was transformed into a 70’s disco. The participants learned a specific sequence of steps consisting of four elements (basic step, cross step, travolta and mixer) and practiced these in time with the music.

After both VR sessions, BP was measured immediately after the session and after five as well as 10 min. This was done three times in total at each time point. In addition, the Borg’s RPE scale [30] and NASA TXL (Task Load Index) [31] were performed. Additional evaluations were conducted after each session which are part of other publications [32–35].

### Materials

The VR high fidelity prototype was part of the development during the BewARe project. The goal of the project was to develop a virtual sensor-based exercise training program for older adults with hypertension. At the time of the study, the prototype was still under development. More details about the gamification concept can be found in a previous publication [32].

An HTC Vive Pro™ VR headset was applied for the study. To enable interactivity five HTC Vive ™ Trackers 2.0 and Valve Index™ controllers were used.

The Polar M600™ smartwatch was used as a monitoring tool to record the participant’s HR during the entire VR exergame. The real-time HR was displayed to the participants in the VR environment via an interface to the VR application. The background of the virtual smartwatch changed the color according to the intensity (underload = blue, individual moderate load intensity = green and overload = red) and can be used for training control. Therefore, framework data (age, height, weight, resting heart rate) were recorded a priori to determine the individual moderate load intensity zone. Existing studies show that the Polar M600 can be used as a valid measurement tool [36, 37].

### Statistical methods

#### Data preparation

The basis for the determination of the load intensity of the participants was the formula of Sally Edwards to calculate the maximum HR (men: HRmax = 214—0.5 × age—0.11 × bodyweight in kg; women: HRmax = 210—0.5 × age—0.11 × bodyweight in kg) [38]. Subsequently, the difference between the maximum HR and the resting HR (HRR) was used to determine the individual moderate load intensity from 40 to 60% for each participant using the Karvonen formula (HRtraining = ((HRmax-HRresting)*intensity%) + HRresting) [39]. In addition, the values of Borg’s RPE scale (value range from 6 to 20 points) were converted into HR values (60 to 200 bpm) for comparison with the objectively measured HR via the Polar M600 [30, 40].

#### Data analysis

Was performed by descriptive and inductive statistical methods using IBM SPSS Statistics 27. The alpha level was set at 5%. For the purposes of inductive statistics, the data were examined for normal distribution using the Shapiro–Wilk test. To test for mean differences, the paired t-test was used under normally distributed conditions. If there was no normal distribution or the data were interval scaled, the Wilcoxon test was used. Repeated measures were tested with ANOVA. Confidence intervals (CI 95%) and effect sizes (d; r) were also provided according to the interpretation of Cohen (1988) [41]. No groups were formed beforehand. Missing value were countered with a listwise case exclusion. A sample size calculation a priori, with a statistical power of 0.80, an effect size of 0.5 and an alpha of 5% (differences between two dependent means, two tails) was performed and resulted in a total sample size of 34.

## Results

### Participants

The results of 22 participants with an age of 74.50 ± 3.64 years were evaluated (Table 2). All participants had essential hypertension as per ICD-10-GM-2021 I10. Most of them were taking at least one of the following drugs: angiotensin II receptor blockers (*n* = 9), anticoagulants (*n* = 7), calcium channel blockers (*n* = 5), ACE inhibitors (*n* = 3), diuretics (*n* = 2), angiotensin-converting enzyme inhibitors (*n* = 1) or β-acetyldigoxin (*n* = 1). Five participants were taking β-blockers. Three participants were not using any medication for the treatment of hypertension. Ten of the participants had previous VR experience. Sixteen participants reported being active in sports more than once a week. Six participants were active in sports less than once a week. On average, the participants participated in sports 2.5 times per week. None of the participants had cognitive impairments, measured by the TICS (37.27 ± 2.57 points), or an increased risk of falling, measured by the Tinetti test (27.64 ± 0.79 points).

**Table 2:** Characteristics of the sample

n	22			
Gender	13 female / 9 male			
	min	max	M	SD
Age	65.00	80.00	74.50	3.64
Height, m	1.54	1.85	1.71	.10
Weight, kg	60.00	103.00	76.18	12.54
BMI, kg/m^2^	20.75	32.51	26.10	3.52
HRresting, bpm	51.00	85.00	63.55	7.62
HRmax, bpm	161.87	173.69	166.01	2.54
HR40%, bpm	95.35	117.47	104.53	4.86
HR60%, bpm	117.52	133.70	125.02	3.64

*min* minimum, *max* maximum, *M* mean, *SD* standard deviation, *BMI* Body Mass Index, *HR* heart rate

### Load intensity

Significant differences between the two VR sessions were found in the mean value of the “main part” and at the “end” as well as for the maximum HR (Table 3).

**Table 3:** Mean HR values of the VR-SET and VR-ET

	VR-SET M (SD), bpm *n* = 21	VR-ET M (SD), bpm *n* = 21	*p*-value	95% CI	d
Start	86.14 (14.12)	83.05 (14.27)	.117	-.84; 7.03	.358
Warm-up	94.01 (16.99)	93.04 (14.02)	.686	-3.93; 5.86	.090
Main part	106.08 (19.70)	96.98 (15.16)	.000***	4.84; 13.36	.973
Cool down	104.42 (20.25)	103.51 (15.64)	.687	-3.76; 5.57	.089
End	100.67 (19.73)	93.33 (15.05)	.002**	3.14; 11.52	.797
HRmax	124.90 (20.65)	114.48 (14.34)	.000***	5.93; 14.93	1.055

*M* mean, *SD* standard deviation, *CI* confidence interval, paired t-test

^**^p < 0.01

^***^p < 0.001

**Fig. 6:**
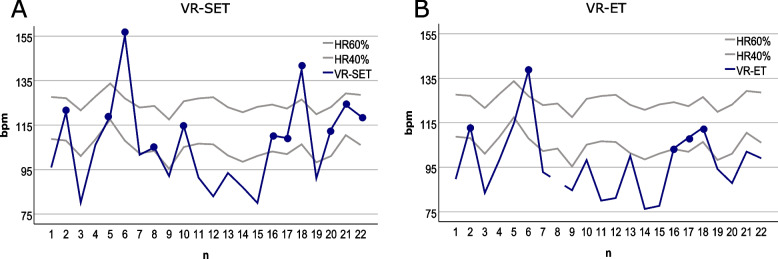
Progression of mean HR during the”main part” in VR-SET (**A**) and VR-ET (**B**) per participant. The black circles indicate exceeding an individual load intensity of 40%. Participant 8 in Fig. **B** is a missing value

A comparison between the VR-SET and the VR-ET showed that significantly more participants reached the desired exercise intensity of 40–60% in the “main part” of the VR-SET than in the VR-ET (*n* = 21; Wilcoxon test *p* = 0.014; *r* = -0.534). In the “main part” of the VR-SET, half of the participants reached their personal intensity range of at least 40% (lower limit of the moderate target zone) (Fig. 6(A)). Because no significant difference could be determined between the 40% limit (M = 104.53 ± 4.56) and the mean of the "main part" (*n* = 22; M = 106.06 ± 19.22; paired t-test *p* = 0.688; 95% CI [-9.15, 6.16]; d = -0.087), participants thus trained at a mean intensity of 40%,In contrast, in the VR-ET, only five participants (27.73%) managed to reach a load intensity of at least 40% (Fig. 6(B)). Consequently, significantly lower load values were achieved in the “main part” of the VR-ET (*n* = 21; M = 96.98 ± 15.16; paired t-test *p* = 0.017; 95% CI [1.53, 13.68]; d = 0.570).

**Fig. 7:**
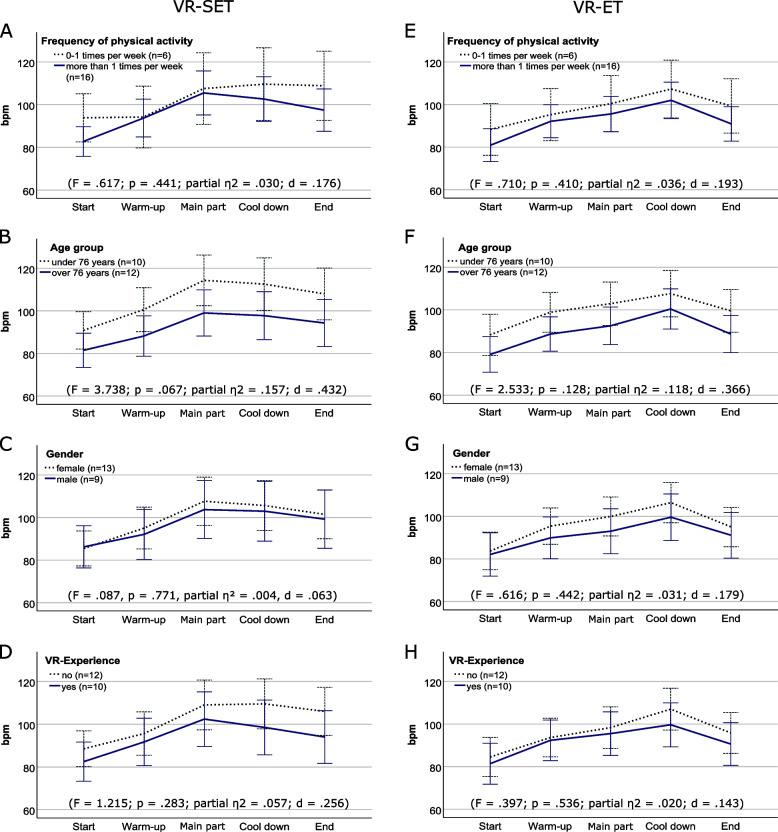
Main Effects of ANOVA with repeated measures for groups in VR-SET and VR-ET

In addition, various group comparisons were made to assess the load intensity during the different training sections. However, ANOVA with repeated measures found no statistically significant main effects between the groups for either VR exergame (Fig. 7(A)-(H)). Only descriptive differences could be determined.

### Perceived exertion

The RPE ranged from "easy" to "somewhat difficult" in the VR-SET (M = 12.14 ± 1.81) and "easy" in the VR-ET (M = 10.82 ± 2.69). For this a statistically significant difference between the two VR sessions were found (*n* = 22; paired t-test *p* = 0.028; CI 95% [1.54, 24.82]; d = 0.502).

When comparing the converted RPE values with the actual measured HR values during the “main part”, the values of RPE were higher than those of the objective measurement in both visits (Table 4). A significantly higher subjective perceived exertion compared to the objectively determined exertion from the HR was found during the “main part” of the VR-SET. With regard to the VR-ET, there were no differences between subjective perception and objective measurement.

**Table 4:** Comparison of subjective perceived and objectively measured load

	Borg Scale M (SD), bpm	HR M (SD), bpm	*p*-value	95% CI	d
VR-SET (*n* = 22)	121.36 (18.07)	106.03 (19.22)	.010**	-26.62; -4.05	-.602
VR-ET (*n* = 21)	110.48 (25.19)	96.98 (15.16)	.052	-27.15; .15	-.450

*M* mean, *SD* standard deviation, *HR* heart rate, *CI* confidence interval, paired t-test

^**^p < 0.01

### Blood pressure

**Fig. 8:**
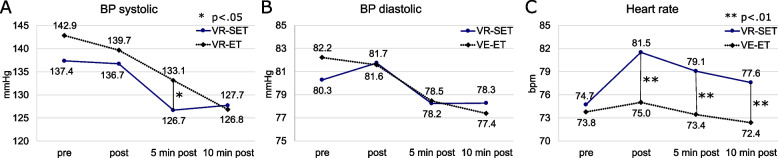
Progression of systolic (**A**) and diastolic (**B**) BP as well as HR (**C**) at the respective measurement times

Over the course of the three postexercise measurements (“post”, “5 min post” and “10 min post”), systolic BP decreased for both the VR-SET and the VR-ET (Fig. 8(A)). In the VR-SET, the tests of within-subjects effects revealed a significant main effect of the exergame on systolic BP (ANOVA with repeated measures, Greenhouse–Geisser correction; *p* < 0.001; partial η^2^ = 0.343; d = 0.722). When systolic BP was measured before and after VR-ET, similar results were obtained (ANOVA with repeated measures, Sphericity assumed; *p* < 0.001; partial η^2^ = 0.551; d = 1.108). The pairwise comparisons between the time points of eachVR session can be seen in Table 5. Moreover, 5 min after the VR session, the systolic BP was significantly lower in the case of the VR-SET than the VR-ET (paired t-test *p* = 0.020, CI 95% [-11.80, -2.51]; d = 0.535). When comparing active and inactive participants regarding systolic BP, there were no interaction between systolic BP and frequency of physical activity in both VR sessions, VR-SET: Greenhouse–Geisser F(43.70, 4026.09) = 0.22, *p* = 0.799, partial η^2^ = 0.011; VR-ET: Greenhouse–Geisser *F*(71.31, 2672.29) = 0.53, *p* = 0.630, partial η^2^ = 0.026.

**Table 5:** Pairwise comparisons of systolic BP in VR-SET and VR-ET

(t1)	(t2)	MD (t1-t2)	SD	*p*-value	95% CI
**VR-SET (*n* = 22)**					
pre	post	.53	3.02	1.000	-8.25; 9.31
	5 min post	10.57	3.07	.015*	1.62; 19,51
	10 min post	9.55	2.85	.018*	1.27; 17,84
post	5 min post	10.04	1.57	.000***	5.48; 14,60
	10 min post	9.02	1.89	.001**	3.52; 14,53
5 min post	10 min post	-1.02	1.61	1.000	-5.69; 3.66
**VR-ET (*n* = 22)**					
pre	post	3.21	1.98	.718	-2.55; 8.98
	5 min post	9.75	2.35	.003**	2.89; 16.61
	10 min post	16.07	2.34	.000***	9.24; 22.89
post	5 min post	6.54	1.52	.002**	2.12; 10.96
	10 min post	12.86	1.87	.000***	7.43; 18.29
5 min post	10 min post	6.32	1.74	.009**	1.26; 11.38

*t* time point, *MD* mean difference, *SD* standard deviation, *CI* confidence interval, ANOVA with repeated measures

^*^p < 0,05

^**^p < 0,01

^***^p < 0,001

For diastolic BP, a decrease in BP values after the VR session was noticeable in both exergames (Fig. 8(B)). Although significant, within-subject effects were determined for the VR-SET (ANOVA with repeated measures, Sphericity assumed; *p* = 0.046; partial η^2^ = 0.118; d = 0.366) and VR-ET (ANOVA with repeated measures, Greenhouse–Geisser correction; *p* = 0.002; partial η^2^ = 0.203; d = 0.505). A significant difference between the time point "post" and "10 min post" was only detected for the VR-ET in the pairwise comparison (MD = 4.19 ± 1.30; *p* = 0.025; CI 95% [0.40, 7.98]).

### Heart rate

However, within the VR-ET, the HR was, on average, almost constant between the time points (Fig. 8(C)) and thus no significant, within-subject effects could be recorded (ANOVA with repeated measures, Greenhouse–Geisser correction; *p* = 0.079; partial η^2^ = 0.101; d = 0.335), in the case of the VR-SET, internal significant main effects were found (ANOVA with repeated measures, Greenhouse–Geisser correction; *p* < 0.001; partial η^2^ = 0.314; d = 0.677). The pairwise comparison for the VR-SET showed differences between the time points "pre" and "post" (MD = -6.80 ± 1.65; *p* = 0.003; CI 95% [-11.62, -1.99]) as well as "post" and "10 min post" (MD = 3.93 ± 1,24; *p* = 0.028; CI 95% [0.32, 7,54]).There were also significant differences between the VR sessions at the time points "post" (paired t-test, *p* = 0.004, CI 95% [3.34, 10.66]; d = 0.693), "5 min post" (paired t-test, *p* = 0.001, CI 95% [2.60, 8.69]; d = 0.821) and "10 min post" (paired t-test, *p* = 0.002, CI 95% [2.15, 8.27]; d = 0.752).

### Perceived task load

**Fig. 9:**
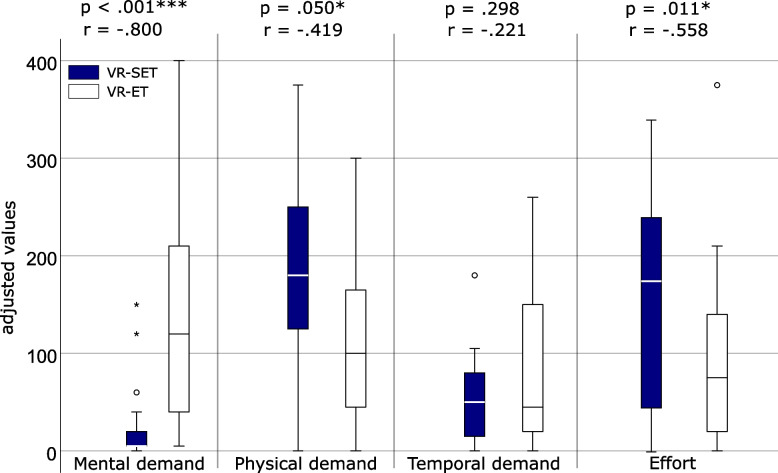
Boxplot of the adjusted task load values of the VR-SET and VR-ET

In terms of the perceived task load (*n* = 22, Wilcoxon test) during the VR exergame (Fig. 9), mental demands were significantly higher in the VR-ET than in the VR-SET. In contrast, the VR-SET imposed significantly higher physical demands. No differences were found with respect to temporal demands. The effort expended to complete the tasks/exercises was significantly higher in the VR-SET than in the VR-ET.

## Discussion

The first purpose of the study was to investigate whether the intensity of the VR exergame was appropriate for older patients with hypertension to exercise within their individually calculated moderate load intensity of 40% to 60% during the VR exergame.

It was determined that a higher load intensity was achieved in the VR-SET than in the VR-ET. In addition, significantly more participants reached the individual moderate exercise zone of 40 to 60% in the “main part” of the VR-SET. In relation to the main question, it can be seen that individuals with the developed VR exergame had limited ability to train in their individual moderate load intensity. The reasons for this could be that the non-personalized number of exercises, the number of repetitions and the number of sets performed are not equally appropriate for all participants to achieve moderate training loads. The speed of exercise execution and breaks, due to initially long exercise explanations, may also have led to a reduction in load intensity. Especially in the case of endurance training, the exercise explanations in the "main part" were significantly longer than for the strength endurance training, which might have led to different load intensities between the two VR sessions. In addition, there were two to three participants who were unable to perform each exercise accurately due to shoulder movement limitations, which may also have resulted in lower loading intensities. In other studies that collected HR measurements during the exergame with older participants, similar [42] or even lower [13] mean values were found compared with the results of this study. Research by Vorwerg et al. [43] found that approximately 65% of the participants of cardiac rehabilitation groups trained within the individual training zone of 50 to 70% [43]. The results illustrate that more individuals in conventional rehabilitation sports groups achieved individual load intensity than in this study. Gonçalves et al., on the other hand, found that participants spent more time participating in moderate to vigorous physical activity during an exergame than during a conventional workout [44]. For the development of the BewARe VR exergame, it can be stated that further personalized adjustments regarding training time, number of exercises, repetitions and sets as well as different dumbbell weight or less explanation time must be implemented in the system. These adjustments should help increase the proportion of people who train within the individual load intensity. The importance of a personalized and adapted training interventions is also suggested by other exergame studies for older adults [45, 46].

The second aim was to evaluate whether VR exergames can have an impact on BP reduction among elderly patients with hypertension. In both VR sessions, systolic BP was significantly reduced after training. Significant main effects were also recorded for diastolic BP. Despite the determined lower load intensity of the participants, a positive influence on BP could be found, which was characterized by a postexercise hypotension for both VR sessions. Higher mean differences were found for the VR-ET, especially for the time point 10 min post. Thus, the VR-ET displayed a stronger post-exercise hypotension. This clinically relevant phenomenon has already been studied numerous times and could also be found in conventional training methods for endurance or strength endurance [47, 48]. However, this phenomenon is exclusively an acute BP reduction.

With regard to the RPE, the present values almost corresponded to the conditions of Löllgen's recommendations or the ACSM exercise guidelines for adults with hypertension, which define moderate intensity as 11–13 on the Borg scale [29, 49]. However, as highlighted by Ciolac et al. in their publication, it should be considered that an evaluation of the RPE after a single training session does not prove the statement that long-term training is perceived as equally strenuous [50]. In addition, it must be noted that the training program may influence the RPE. Accordingly, in this study, lower RPE values were found for VR-ET than for VR-SET. These results are also reflected in the perceived task load, in which higher physical demands were recorded for the VR-SET. In particular, Morishita et al. maintain that in relation to strength training for the elderly, the Borg 6–20 RPE is a useful assessment for elderly individuals who perform resistance training [51]. However, training using the Borg scale can also lead to overload in patients with cardiovascular disease [52]. This could be a reason for the participants having a subjectively higher perceived exertion compared to the measured HR.

Another point is that according to the recommendations of cardiac societies, persons with hypertension should participate in endurance training five times a week for 30 min [8–10]. As is also evident in this sample, older individuals are physically active on average two to three times per week. The gap between recommendations and the actual physical activity completed, was also described by Rhodes and de Bruijn in their publication [53]. To provide an additional incentive, VR exergames could help increase motivation for additional training sessions and simultaneously achieve health-promoting effects [13, 54].

### Strength and limitations

A strength of the study is that the comparability of HR and BP between the two VR sessions is very high due to the identical study population.

However, there are also several limitations. Unfortunately, the calculated sample size could not be met due to the refusal of certain potential participants to take part in the study as a result of the COVID-19 pandemic.

The study's main limitation is the absence of a control group. Without a control group or session for comparison, it is challenging to attribute the observed changes in the dependent variables solely to the experimental treatment. This absence introduces potential threats to internal validity, such as maturation and selection bias. Despite this limitation, the study provides valuable insights into the effects of the treatment within the VR sessions.

Another limitation of the study is the lack of randomization of the two VR sessions. Although each participant had the VR-SET in the first week (visit 1) and the VR-ET in the second week (visit 2), the order of the VR sessions may still have had an influence on the perception of them. The order of the sessions could potentially confound the results as the experience gained influences the behavior of the participants during the second session. By not employing randomization, the study may be prone to allocation bias.

One further limitation was the lack of blinding in the study. Blinding of studies in physical and rehabilitation medicine is particularly complex compared to other areas of medicine [55, 56]. The meta-analysis of Armijo-Olivo et al. [57] showed that trials with inappropriate blinding of assessors and participants in physical therapy tended to underestimate treatment effects, the difference was not statistically significant to appropriate blinding. However, the authors emphasize that it does not mean that there is no effect. Armijo-Olivo et al. [57] recommend researchers to look for creative solutions to avoid performance and detection bias when possible.

Furthermore, there are recommendations for the determination of postexercise hypotension, which state that a measurement should be taken at least up to 20 min, or even better 120 min after training, to assess the effect [47]. The BP measurements in this study were only taken up to 10 min after VR session, which reduces the information regarding existing postexercise hypotension. Greater clinical relevance is also associated with a consideration of blood pressure in a 24-h context [58].

In addition, at this stage of the project, the VR exergame was not yet completely developed, which meant that the ACSM recommendations of a minimum of 30 min of aerobic exercise or moderate-intensity strength exercise [29] could not be satisfied for a VR session.

Another point of concern relates to the individuals chosen to take part in the VR sessions, which could have an impact on HR and BP. Conjectures suggest that older people might be afraid or excited due to the low acceptance of VR technologies. However, the results from other publications suggest that VR applications with regard to the health of older adults are not affected by negative attitudes [59].

A further significant limitation in the consideration of the load intensity and BP relates to the medication. In the evaluation of the results, a more detailed consideration of the influence was omitted. However, as described, β-blockers and angiotensin II receptor blockers, among others, lead to a reduced HR at rest as well as during exercise [29]. There was no instruction on standardized intake.

Although the results of the Simulator Sickness Questionnaire (SSQ) [34] ruled out the presence of severe symptoms of VR sickness in this regard, the wearing of a VR headset during training may have led to an inhibition of movement action and thus to a lower load intensity. Due to the short intervention period of study dropouts with VR sickness symptoms, it was not possible to clarify how load intensity would relate to these symptoms. Stamm et al. [34] indicated that participants tended to have a higher SSQ total score in the VR-ET than in the VR-SET. The authors suspected increased movement in space as a cause for this. Significant differences were only observed in oculomotor symptoms (subscale), which were higher in VR-ET.

Furthermore short signal interferences of the VR headset, which resulted in additional breaks, could lower the load intensity. Even though the Polar M600 is considered suitable, erroneous measurements cannot be excluded. However, to preserve internal validity, the same watch was always used.

### Implications for further research

Our future research should address the limitation of the absence of a control group by incorporating a control group to strengthen internal validity and enhance the study's overall robustness. To strengthen future research endeavors, we recommend the inclusion of a control group in subsequent studies. The integration of a control group will allow for more robust statistical comparisons and a more accurate assessment of the intervention's effects. In addition, randomization and blinding should be provided for future studies.

Furthermore, the impact on HR and BP could not be proven due to various influencing factors such as VR experience, sex, age group, training frequency and medication intake on physiological and perceptual responses. Descriptive differences show that these factors should be investigated in further studies. To enable this, a larger sample and regression models should be used. In order to derive a greater clinical relevance, a consideration of the blood pressure in the 24-h context would be desirable and should be considered in subsequent investigations.

Moreover, the further development of the exergame requires an adjustment of the training duration in order to follow the ACSM recommendations of at least 30 min training on the one hand and to increase the training intensity on the other hand. This can also be achieved through the number of exercises, repetitions and sets as well as different dumbbell weight.

## Conclusion

Considering the importance of increasing hypertension prevalence in old age and the lower physical activity of older people, our results show that a VR exergame could lead to blood pressure lowering effects for older people with hypertension. Accordingly, as with conventional training methods, postexercise hypotension was observed for both VR sessions in our study. However, our results also showed that, on average, less than half of the participants trained within their individual moderate load intensities. Moreover, the findings obtained can serve as a basis for further studies and have, therefore, focused for the first time on a target group of patients with hypertension. However, future studies need to examine the long-term benefits of VR exergame training for elderly patients with hypertension.

## Data Availability

The datasets used and/or analysed during the current study are available from the corresponding author on reasonable request.

## Abbreviations

ACC: American College of Cardiology
ACSM: American College of Sport Medicine
AHA: American Heart Association
BMI: Body mass index
BP: Blood pressure
CVD: Cardiovascular diseases
CI: Confidence interval
ESC: European Society of Cardiology
ESH: European Society of Hypertension
HR: Heart rate
HRR: Heart rate reserve
ISH: International Society of Hypertension
M: Mean
max: Maximum
MD: Mean difference
min: Minimum
η2: Eta-Quadrat
RCTs: Randomized controlled trials
RPE: Rating of Perceived Exertion
SD: Standard deviation
SSQ: Simulator Sickness Questionnaire
TICS: Telephone Interview for Cognitive Status
VR: Virtual reality
VR-SET: VR strength endurance training
VR-ET: VR endurance training
